# Using Technology to Nurture Core Human Values in Healthcare

**DOI:** 10.15694/mep.2019.000223.1

**Published:** 2019-12-05

**Authors:** Poh-Sun Goh, John Sandars

**Affiliations:** 1 National University of Singapore; 2 Edge Hill University Medical School

**Keywords:** Technology to nurture values, Narratives to nurture values, Technology for narratives, Virtual Reality to nurture values

## Abstract

This article was migrated. The article was marked as recommended.

The important task of nurturing core human values in healthcare can be assisted and facilitated by the use of a variety of technology, to create, store, and disseminate a range of narratives in health and illness, from text and video to immersive experiences using virtual reality. This paper will discuss the importance of narratives to nurture core human values, the use of technology for narrative to nurture core human values, as well as the challenge of using technology for narrative to nurture core human values.

## Introduction

The International Charter for Human Values in Healthcare presents five core values that are essential for effective healthcare ( Rider *et al.,* 2014): compassion, respect for persons, commitment to integrity and ethical practice, commitment to excellence and justice in healthcare. Developing and sustaining these values across the lifelong journey of becoming and being a doctor is essential, and the ubiquitous presence of technology is increasingly having a central role in nurturing these core values.

Narratives are essential to nurture values in healthcare since they promote self-reflection ( Chou *et al.,* 2014), and technology is becoming increasingly important for the creation, storage, and dissemination of a wide range of different narratives, from text and video to immersive experiences using virtual reality (VR). In this article we will highlight the potential of using a variety of technology for nurturing core values through self-reflection both in the creation and consumption of different narratives.

## The importance of narrative to nurture core values

A narrative is “anything that tells or presents a story” and all narratives have several features ( Jahn, 2017), including the topic and a medium to present the story, such as a podcast or video. The most important feature of a story is an intended reaction in members of the audience, such as a feeling of surprise or sadness. Dewey (1934) highlighted the importance of ‘having an experience’ that occurs when presented with a narrative, especially when this experience is an unexpected emotional reaction. When faced with this reaction, the person tries to make sense of why they have had the reaction by engaging in self-reflection. Often the reaction is because the main beliefs and core values of the person are challenged by the experience. The process of self-reflection is particularly enhanced when it is guided by another person, such as an educator or peer, and also when there is a cycle in which there is an opportunity to put new insights into action, which leads to further self-reflection ( Dewey, 1933).

We are inspired and moved by narratives. Professional practice in healthcare is replete with a wide range of different experiences and narratives, including suffering, comfort, inspiration and joy. Narratives can be told from the perspective of patients, relatives, caregivers, healthcare practitioners and wider members of the healthcare team. These narratives can be in written form, be told or recounted orally, performed, observed in person or from a video.

Consumption of narratives are a powerful prompt for self-reflection. “An example of a narrative which gives insight into how a professional in the palliative care sector-practice might approach dying patients was recently published in the New York Times ( Puri, 2019). Similarly, the ending scene from the 1991 movie ‘The Doctor’ illustrates how an initially insensitive, emotionally distant medical practitioner develops a very personal insight into the perspective of the healthcare system when he himself became a patient, and was motivated to introduce a patient centred experience for medical students in his medical school’s medical program. The book ‘A taste of my own medicine: When the doctor is the patient’ by Edward E Rosenbaum in 1991 narrates a similar story.” ( Goh, 2019).

It is important to remember that creating narratives can also be powerful in the promotion of self-reflection ( Baron and Pletsch, 2019), and similarly so can self-reflection through telling narratives to peers ( Player *et al.,* 2019).

“Narratives can encourage all of us to self-reflect on our roles in the promotion of health and managing disease as healthcare practitioners, helping us to improve our empathy, communication skills, teamwork, organisational abilities and professionalism. We can become more sensitive to the point of view of a patient or caregiver. We can become better at demonstrating that we care through improved communication, with more skill and sensitivity. Giving attention to the essential human side of medicine complements the scientific and technical side of medical practice.” ( Goh, 2019).

## The importance of technology for narrative to nurture core values

Technology has an increasing part in the creation, storage and dissemination of stories and narratives ( Goh, 2016). “There are opportunities to scale up our educational and training efforts, to engage students and practitioners beyond the classroom and to be used before, during and after traditional classroom training. We can use open source content, and low cost-free platforms to communicate with, and engage our students, and fellow practitioners. Technology makes it easier for us to create and curate content. And share this content through websites, blogs and apps. Mobile devices with free or low cost easy to use software and apps make it relatively simple and easy to create and edit, or curate text, illustrations, audio, multimedia, and video content.” ( Goh, 2019).

Widely available low cost technology like websites and blogs can be used to provide access to, and disseminate patient stories and narratives, as well as to facilitate and support guided reflection and discussion. Websites and videos can illustrate and showcase illness stories and narratives.

Virtual reality (VR) and immersive reality experiences can bring an online audience remotely into a practice setting and simulate the experience of illness for students and healthcare practitioners ( Dyer *et al.*, 2018). These newer technologies increasingly simulate real life settings and are more immersive, deepening our insights into a patient’s point of view ( Herrera *et al.*, 2018). Artificial Intelligence (AI), can adapt and personalise the content to provide a personalised interactive digital experience, with the potential to increase empathy and compassion ( Lakhani, 2019). While AI and VR are high cost methods to use technology to build empathy, lower cost solutions including mobile apps to build empathy have also been developed ( Konrath, 2017; Papoutsi and Drigas 2017). We can therefore consider a spectrum of realism to abstraction - from real life to VR, interactive theatre, video and multimedia, audio narratives and podcasts, illustrations and photos, to written narratives.

## Examples of technology with narrative to nurture core values

**Illustrations and Art** - the traditions of art, illustrations and photography to capture scenes of illness and healthcare can broaden the exposure and experience of healthcare practitioners to situations and points of view

Visualising Illness ( Biernoff and Johnstone, 2017).

http://www.bbk.ac.uk/art-history/research/visualising-illness

and

Psychiatry student uses art to shed light on the darkest shades of illness ( McMaster, 2018).

https://www.folio.ca/psychiatry-student-uses-art-to-shed-light-on-the-darkest-shades-of-illness/

**Video** - is a powerful visual and auditory medium that can be used to depict health and illness, and be easily shared on multiple mobile and online platforms

Facing depression: Ep 4: An Elderly’s Perspective ( Channel News Asia, 2018).

https://www.channelnewsasia.com/news/video-on-demand/facing-depression/an-elderly-s-perspective-10036596

**Podcasts** - take advantage of the qualities of the human voice and sounds to convey emotion and feeling, to move the listener

How Podcasts Make Me an Empathetic Physician ( McFarlane, 2018).

https://blogs.bmj.com/medical-humanities/2018/05/01/how-podcasts-make-me-an-empathetic-physician/

and

“Your Stories” Podcasts: How Cancer Impacts Families ( Wroten, 2018).

https://www.cancer.net/blog/2018-03/your-stories-podcasts-how-cancer-impacts-families

**Video games** - interactive video, online and mobile games can be designed as guided reflective exercises, to build empathy ( Kral *et al.,* 2018)

Can A Video Game Boost Empathy in Teens? ( Pedersen, 2018).

https://psychcentral.com/news/2018/08/11/can-a-video-game-boost-empathy-in-teens/137794.html

## The challenge of using technology for narrative to nurture core values

“As with all use of technology to enhance and augment our educational and training efforts, these efforts should be driven first by our instructional and educational objectives and not the technology.” ( Goh, 2019). The key ingredient in the use of narratives to build empathy is by highlighting a patient’s point of view ( Milota *et al.*, 2019), but this needs to be combined with opportunities to have purposeful guided reflection on the core messages that the narrative can offer ( Laughey *et al.*, 2019). The additional ingredient of feedback on professional behaviour and communication approaches with real and simulated patients is a particularly powerful learning opportunity ( Laughey *et al.*, 2019).

There are already a vast range of different narratives that have been produced and with the potential to nurture core values. However, a current challenge is that this enormous resource is widely dispersed and cannot be easily accessed by potential users. A priority is the concerted effort to systematically document and curate these narratives through online digital repositories.

An important further consideration for all educators is the increasing awareness of “digital empathy”. Medical students and healthcare practitioners will need to develop their understanding of how digital tools and communication platforms reduce the available communication cues and signals that are traditionally present in face to face communication to convey feelings and emotions, and express empathy and compassion ( Terry and Cain, 2016).

Finally, as educators, content creators and curators, we will need to develop our experience and skills in design thinking for empathy ( Dam and Teo, 2018), and “empathic design” ( Wang and Huang, 2010), and collaborate with professionals with these skills. The empathic design process infuses empathy and awareness of individual perspectives throughout the whole design process, from conception to implementation, and requires constant self-reflection on how the educator makes the crucial decisions about the learning needs of learner, the choice of content and technology, and the potential impact on the learner. The idea of empathic design can also be taken further in our planning and delivery of a holistic patient experience, beyond the human interactions to the practice and healthcare setting and healthcare delivery process ( Freihoefer, 2018).

## Take Home Messages

A variety of technologies can be used to nurture core human values in healthcare. Technology is becoming increasingly important for the creation, storage, and dissemination of a wide range of different narratives, from text and video to immersive experiences using virtual reality. In addition to an awareness of the range of available technology, as well as spectrum of digital formats of narratives in health and illness, educators will need an awareness of and skills in digital empathy, design thinking and empathic design; and to collaborate with professionals with these skills.

## Notes On Contributors

Poh Sun Goh, MBBS, FRCR, FAMS, MHPE, FAMEE, is an Associate Professor and Senior Consultant Radiologist at the Yong Loo Lin School of Medicine, National University of Singapore, and National University Hospital, Singapore. He is a graduate of the Maastricht MHPE program, a member of the AMEE TEL committee, and a Fellow of AMEE. ORCID: https://orcid.org/0000-0002-1531-2053

John Sandars MB ChB (Hons), MSC, MD, MRCP, MRCGP, FAcadMEd, is Professor of Medical Education at Edge Hill University Medical School, Ormskirk, UK, and is Co-Chair of the AMEE Technology Enhanced Learning Committee. ORCID: https://orcid.org/0000-0003-3930-387X
