# Supplementary material for: Using Technology to Nurture Core Human Values in Healthcare
Source: MedEdPublish (2016). 2019 Dec 5;8:223. [Version 1] doi: 10.15694/mep.2019.000223.1 (PMC10712449; doi:10.15694/mep.2019.000223.1)
Supplement: Peer review history

# Reviewer report: Reviewer response for version 1

**DOI:** 10.21956/mep.19946.r30288

**Published:** 2019-12-08

**Authors:** P Ravi Shankar

**Affiliations:** 1 American International Medical University

This review has been migrated. The reviewer awarded 4 stars out of 5 This is an interesting paper. The traditional belief is that technology reduces the human element in healthcare. The authors allude to this toward the end of the paper where they briefly touch on digital empathy. I would have liked them to discuss more on this important topic which is not adequately discussed. Online resources make it easy for teachers and students to access different resources. This is especially true for institutions which do not have big libraries and access to museums and other resources. Technology can lead to democratization of access. I have been using material from the New York University Litmed database for the medical and health humanities modules which I facilitate. Technology can help provide access to visual art which is in the public domain in addition to the narratives emphasized by the authors. The article is a personal opinion but I would welcome a more detailed description of different online resources which are available to teachers and facilitators of the medical humanities.

Reviewer Expertise:

NA

No decision status is available

# Reviewer report: Reviewer response for version 1

**DOI:** 10.21956/mep.19946.r30287

**Published:** 2019-12-07

**Authors:** Ken Masters

**Affiliations:** 1 Sultan Qaboos University

This review has been migrated. The reviewer awarded 3 stars out of 5 An interesting opinion piece on using technology to nurture core human values in healthcare. The gist of the argument is two-stage: (1) core human values can be understood through narratives, and (2) narratives can be recorded, stored and disseminated through technology. I particularly liked the inclusion of VR and other more sophisticated technology so that the experiencer of the narrative shifts from being a consumer of the narrative to a co-creator. Perhaps the authors could share a few more examples of these.While the paper does make the main points ((1) and (2) outlined above) clearly, I feel that it falls short of providing the real value to the reader: how to use these in medical education. Education is introduced only briefly in the last section, and then only very generally, saying, essentially, these can and should be used in education. The point is well-made, but the real value would be in answering the question “How?” This is particularly important because the title, with the opening word of “Using” gives the impression that this will be the focus of the paper. So, I would really like to a new section at the end of the paper (or otherwise significantly expanding the current final section) in which the authors explain how they would see the examples used. (E.g. the video on depression is powerful. How would I, as an educator, use this in my teaching? To which students, under which circumstances? What would specific challenges be, etc.) Two minor points:• Given that the Charter is central to the paper, it would have been useful if the authors had linked to the site (http://charterforhealthcarevalues.org/) in addition to the link to Rider et al.• The authors might wish to address (even briefly) the issue of fiction versus nonfiction. I say this because many readers may first associate narratives with first-hand accounts of events, but some of the examples given in the paper are fictional, and one might wonder whether the impact of a narrative is somewhat diminished if it is an entirely fictional account rather than based on real (albeit anonymised) events.(By the way, the authors and readers may be interested in seeing some patient stories shared on YouTube and elsewhere. One in particular, that unfolded over a series of videos and I have found useful in teaching, are those by Simone Giertz beginning with “I have a brain tumor” at https://www.youtube.com/watch?v=tpa4kp4lK60). So, while the authors have a really nice idea, and argue their points well, I would like to see a Version 2 of the paper that has been rounded off with a section on practical descriptions of usage.

Reviewer Expertise:

NA

No decision status is available

# Reviewer report: Reviewer response for version 1

**DOI:** 10.21956/mep.19946.r30285

**Published:** 2019-12-07

**Authors:** sathyanarayanan varadarajan

**Affiliations:** 1 SRM Medical College, SRM University

This review has been migrated. The reviewer awarded 4 stars out of 5 In this interesting article, the authors have nicely highlighted the importance of narrative to nurture core values in healthcare and the potential of using a variety of technology for narrative for nurturing core values. They have discussed these technologies with suitable examples and also described the challenges for using technology for narrative to nurture core values.The authors also highlighted the importance of newer technologies like Virtual reality (VR), Artificial Intelligence (AI) and how they can be of immense help in nurturing the core values like empathy and compassion. The authors also provided appropriate links for examples of technology with narrative such as Illustrations & Art, Video, Podcasts and Video games.This article will be helpful to create awareness of the range of available technologies used to nurture core human values in healthcare to all healthcare practitioners and medical educators involved in teaching core human values. This article is not only interesting but also highly relevant to the need of educating young medical students on core human values through technology.

Reviewer Expertise:

NA

No decision status is available

# Reviewer report: Reviewer response for version 1

**DOI:** 10.21956/mep.19946.r30286

**Published:** 2019-12-06

**Authors:** Jonathan Guckian

**Affiliations:** 1 Leeds Teaching Hospitals Trust

This review has been migrated. The reviewer awarded 4 stars out of 5 Lovely reflections, grounded in scholarship. Particularly enjoyed the discussion of 'digital empathy'. Increasingly relevant in a fast-paced technological era.I would have been interested in the other side of the coin - the potential for technology to impair 'core values' through negative cultures, particularly on social media.

Reviewer Expertise:

NA

No decision status is available
